# Effects of Cold Rolling and Annealing Prior to Dealloying on the Microstructure of Nanoporous Gold

**DOI:** 10.3390/nano8070540

**Published:** 2018-07-18

**Authors:** Hanyu Hui, Re Xia, Juying Li, Qingsong Mei, Ye Ma, Feng Chen, Yan Lei

**Affiliations:** 1 School of Power and Mechanical Engineering, Wuhan University, Wuhan 430072, China; huihanyu0927@163.com (H.H.); xiare@whu.edu.cn (R.X.); mayer@whu.edu.cn (Y.M.); fengc1004@163.com (F.C.); yappee@126.com (Y.L.); 2 Key Laboratory of Hydraulic Machinery Transients, Ministry of Education, Wuhan University, Wuhan 430072, China; 3 School of Mechanical Engineering, Wuhan Polytechnic University, Wuhan 430023, China; jylimei@163.com

**Keywords:** nanoporous gold, microstructure, dealloying, cold rolling, annealing

## Abstract

The properties of nanoporous gold (NPG) were known to be dependent on the microstructure of NPG. In this study, the effects of cold rolling and annealing of the original Ag_0.7_Au_0.3_ alloy on the microstructure of NPG produced by dealloying under free corrosion condition were investigated. Ag_0.7_Au_0.3_ alloy samples were cold-rolled to different strain levels/thickness reductions up to 98% and annealed at 900 °C for 3 h before dealloying. It was found that cold rolling and annealing of the original alloy can lead to reduced ligament and pore sizes of NPG. Moreover, post-deformation annealing of the original alloy was found to facilitate the formation of a homogeneous and continuous NPG structure. The minima of pore and ligament sizes (both being ~8 nm) with uniform distribution were obtained in the annealed sample with a thickness reduction of 60% for a dealloying time of 7 h. The present study indicated the significant effect of a pre-dealloying treatment of the original alloy (by plastic deformation and annealing) on the formation and optimization of the NPG microstructure produced by dealloying.

## 1. Introduction

Due to their chemical stability, strength, and high specific surface area, nanoporous metals have attracted great interests in many applications, including catalysis, fuel cells, sensors, and more [1,2]. Current popular methods for the preparation of nanoporous metals include template methods [3] and dealloying [4]. Nanoporous metals, including Au, Cu, Ni, Ag, and Pt, have been successfully fabricated by chemical or electrochemical dealloying [4,5,6,7,8], among which much attention has been paid to the prototypical Ag–Au system. The dealloying of an Ag–Au alloy can produce nanoporous gold (NPG) with an open, three-dimensional bicontinuous interpenetrating ligament-channel structure with nanometer length scales [4,5,7].

The properties of NPG were found to depend on the microstructure of NPG, such as length scales of ligaments and pores [9,10,11]. Studies have revealed that the sizes of pores and ligaments of NPG produced by a dealloying of Ag–Au alloy were influenced by corrosion time, corrosion temperature, and an annealing of samples after corrosion [12,13,14]. The original Ag–Au alloy was usually prepared in various states before dealloying [15,16,17,18,19,20,21], for which the different fabrication and processing methods and states of the original alloy were found to have significant effects on the morphology of the NPG microstructure [16,17].

Plastic deformation and annealing are common methods used for the processing and microstructure modification of metals. It is well known that plastic deformation can result in an increase of lattice defects and even grain refinement in metals, and a subsequent annealing can lead to the recovery and recrystallization of the deformed structure by formation of more homogenous and refined grains with reduced lattice defects [22,23,24]. Thus, it is expected that plastic deformation and annealing prior to dealloying can have significant effects on the dealloying process and hence the NPG microstructure, for which experimental evidences are still lacking. In this work, the original Ag–Au alloy was subjected to cold rolling and annealing to investigate their effects on the microstructure of NPG produced by subsequent dealloying.

## 2. Experimental

The Ag_0.7_Au_0.3_ (wt. %, purity, 99.99%) alloy ingot used in this study was purchased from China New Metal (Beijing, China). The ingot was cut into 7 × 7 × 1 mm^3^ pieces as original samples. The cold rolling of the Ag_0.7_Au_0.3_ alloy was performed with thickness reductions up to 98% at the speed of 1 rpm (referred to as CR samples). The annealing of cold-rolled samples was carried out at 900 °C for 3 h under argon atmosphere followed by furnace cooling (referred to as AN samples). Samples were polished with abrasive paper and then cleaned with dehydrated alcohol in an ultrasonic cleaning bath before dealloying. The dealloying was performed by immersing samples in a 68% nitric acid solution at room temperature under free corrosion conditions for 3 h or 7 h. The as-dealloyed samples were rinsed repeatedly using dehydrated alcohol before further investigation. The microstructure was observed using a ZEISS SIGMA field emission scanning electron microscope (FE-SEM) (Carl Zeiss Microscopy Ltd, Cambridge, United Kingdom), operated at an accelerating voltage of 5 kV. The pore and ligament sizes, defined as the width and diameter of the pore, as well as the wall thickness of the band, respectively, were averaged by manually measuring about 100 data points of SEM images.

## 3. Results and Discussion

**Figure 1:**
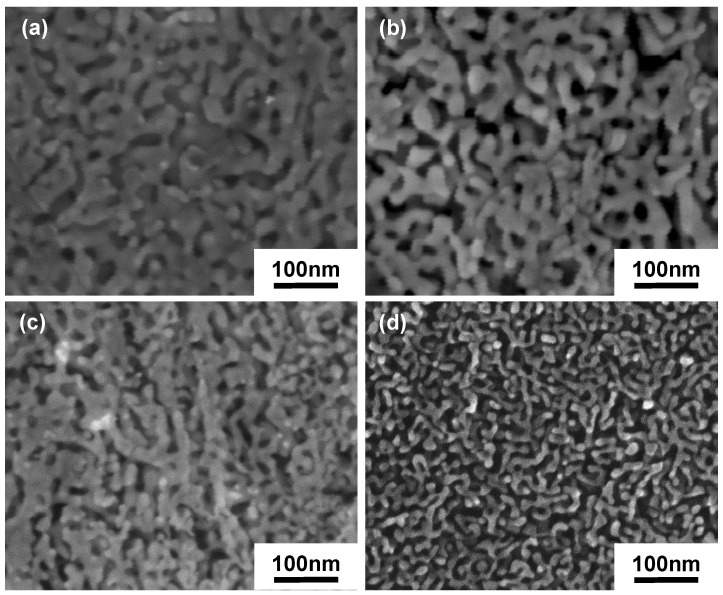
SEM images of NPG produced by dealloying of CR samples with different thickness reductions: (**a**) 0%; (**b**) 60% and (**c**) 90% for a dealloying time of 7 h and (**d**) 98%, for which a shorter dealloying time of 3 h was used to avoid over-corrosion of the sample and to compare the NPG structure of samples with and without post-deformation annealing with the same dealloying time of 3 h.

As shown in Figure 1, after dealloying the CR samples, the NPG structure was produced with average ligament sizes of 8~20 nm, and average pore sizes of 8~16 nm, respectively, depending on the degree of plastic deformation. It was found that both the ligament and pore sizes of NPG produced by the dealloying of the CR samples decrease with increasing thickness reduction, which can be attributed to the increased lattice defects, such as point defects and dislocations in the CR samples induced by plastic deformation. It is known that formation of NPG by dealloying of Ag–Au alloy is due to the selective corrosion of Ag [20,25]. During the dealloying, lattice defects (point defects and dislocations) can act as preferred sites for corrosion. Therefore, an increase of the lattice defects can lead to increased corrosion sites, and hence a reduction in NPG pore size. It is noted that some areas of the NPG samples (Figure 1) are still discontinuous, which is mainly due to the large number of defects caused by cold rolling and the microscopical heterogeneity of plastic strain in the sample.

**Figure 2:**
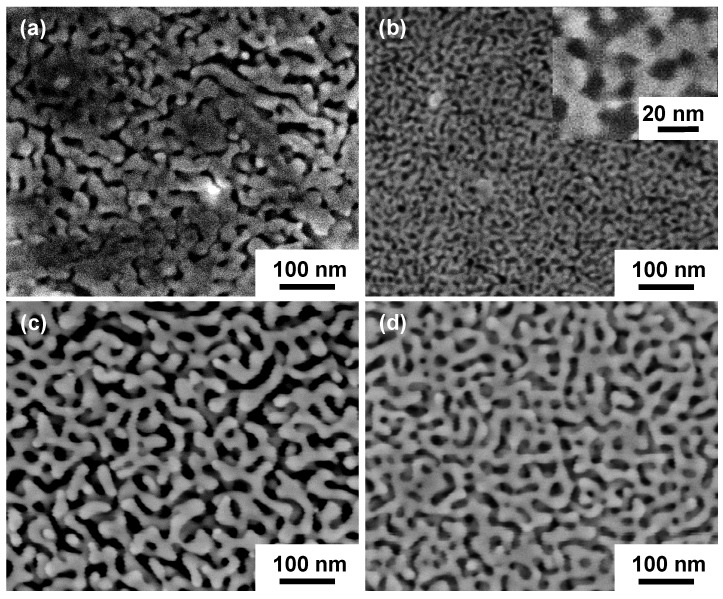
SEM images of NPG produced by dealloying of AN samples with different thickness reductions: (**a**) 0%; (**b**) 60% and (**c**) 90% cold rolled and annealed for a dealloying time of 7 h and (**d**) 98% cold rolled and annealed for a dealloying time of 3 h.

The CR samples were further annealed at 900 °C for 3 h and subjected to dealloying (AN samples). Figure 2 shows the corresponding NPG microstructure for the AN samples. As shown in Figure 2, an open, bicontinuous interpenetrating ligament-channel structure of NPG with an average ligament sizes of 8~20 nm and an average pore sizes of 8~14 nm, respectively, was produced in the AN samples, depending on the thickness reduction of the cold rolling. It is clear that compared with the CR samples (Figure 1), the AN samples have a more uniform and better connected nanoporous structure (Figure 2).

**Figure 3:**
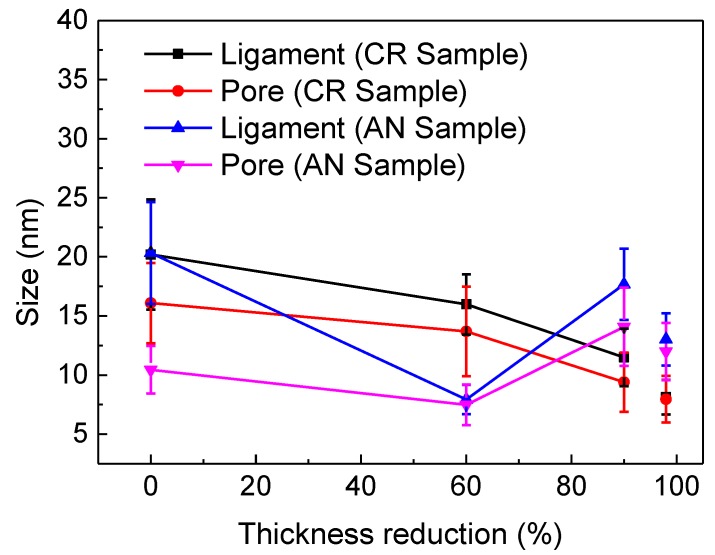
Average ligament and pore sizes of NPG of CR and AN samples as functions of thickness reduction.

Figure 3 depicts the average pore and ligament sizes as functions of thickness reduction for CR and AN samples as indicated. The detailed numerical data are shown in Table 1. As shown in Figure 3, the AN samples have smaller pore and ligament sizes than those of the CR samples with a thickness reduction of 60%, while larger ones with a thickness reduction of 90%, i.e., the minima of pore and ligament sizes (both are ~8 nm) were obtained in AN sample with a thickness reduction of 60% for a dealloying time of 7 h. Similarly, a more uniform and continuous NPG structure is observed in the AN sample than those in the CR sample, with a thickness reduction of 98% and a dealloying time of 3 h under the same free corrosion condition, despite a slight increase of the pore size (~12 nm) in the AN sample compared to that of the CR sample (~8 nm). This can be attributed to the faster corrosion rate in the AN sample with a larger thickness reduction: in the same corrosion time, the structure of NPG is more quickly stabilized, and a single nanopore coarsening stage occurs in the AN sample with a larger thickness reduction. Compared with previous studies of NPG produced by means of a free corrosion method with pore and ligament sizes mostly in the range of ~6–60 nm [4,12,15,20,21,26], the ligaments and pores of NPG produced in this study have almost reached the minima. It should be noted that simply by pre-dealloying deformation and annealing, one may not get the best NPG microstructure over all other methods, since the final formation of NPG can be influenced by many other factors, such as original alloy composition and microstructure, dealloying condition, heat treatment after dealloying, and so on.

**Figure 4:**
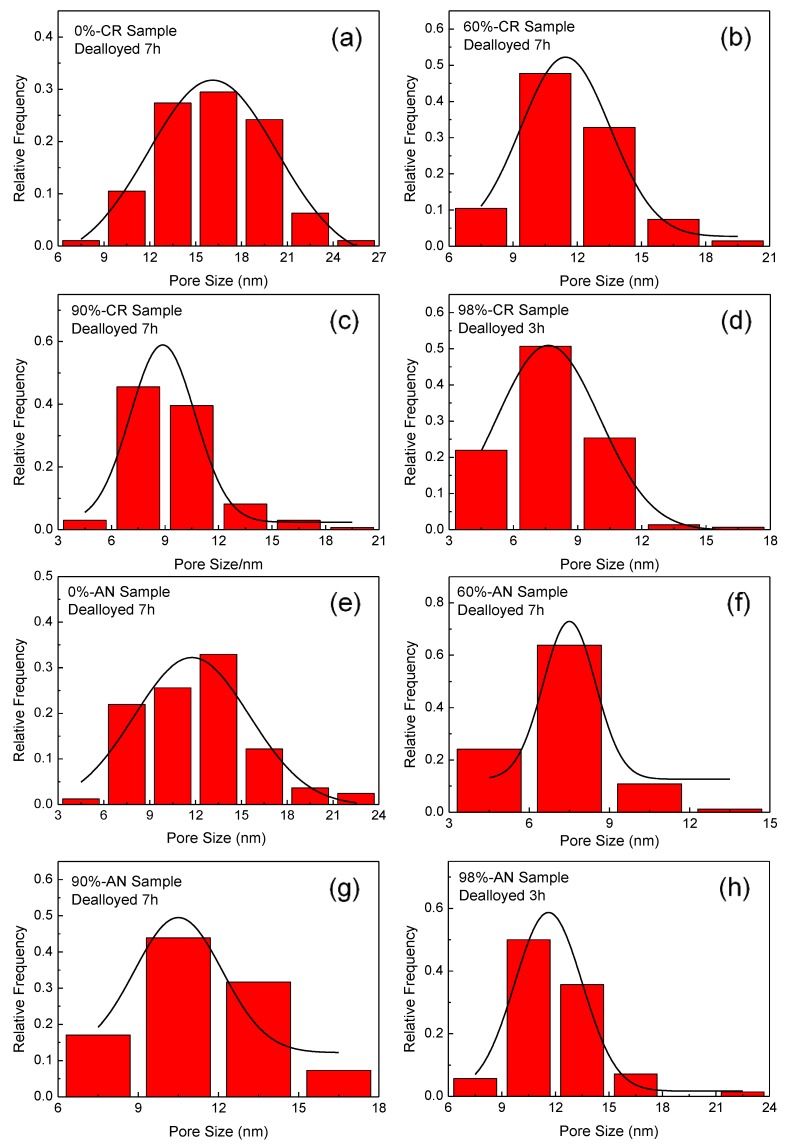
Pore size distribution with corresponding Gauss fit for different samples as indicated. (**a**–**d**) CR samples with different thickness reductions of 0%, 60%, 90% and 98%, respectively; (**e**–**h**) AN samples with different thickness reductions of 0%, 60%, 90% and 98%, respectively.

**Figure 5:**
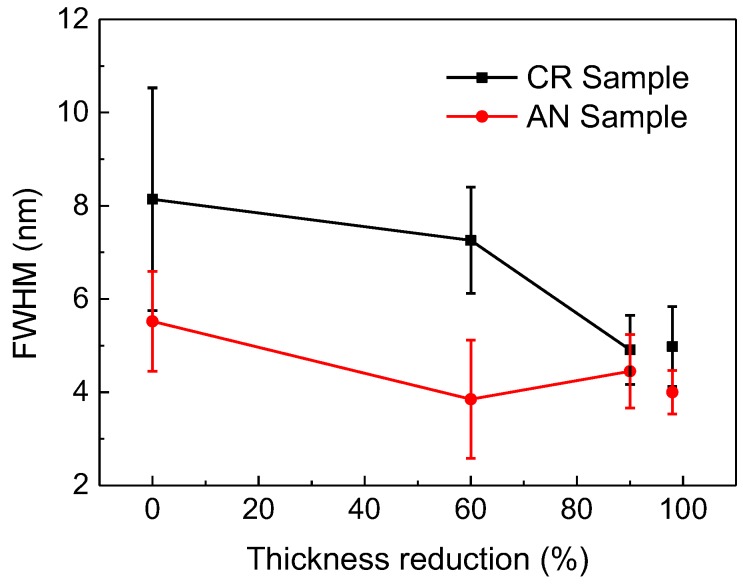
FWHM of the Gauss function fitting curves of the pore size distribution of CR and AN samples as functions of thickness reduction. For the sample with a thickness reduction of 98% a dealloying time of 3 h was used and for the others a dealloying time of 7 h was used.

To further characterize the uniformity of the pores, the full width at half maxima (FWHM) of the Gauss function fitting curves of the pore size distribution were measured, as shown in Figure 4. Naturally, the smaller the value of FWHM, the more uniform the pores of the NPG sample. Figure 5 plots FWHM as a function of thickness reduction for CR and AN samples, as indicated. As shown in Figure 5, the FWHM shows a clear decrease in the CR samples as compared to that of the undeformed sample, indicating a more uniform microstructure of NPG induced by the cold rolling of the original sample. Moreover, one can see from Figure 5 that AN samples show evidently smaller FWHM than those of CR samples, i.e., the distribution of pores is more uniform in AN samples. From Figure 3 and Figure 5, one can see that the AN sample with a thickness reduction of 60% and a dealloying time of 7 h has both the smallest pore and ligament sizes and FWHM, indicating an optimal condition for the production of NPG with refined pore size and uniform distribution by dealloying.

## 4. Conclusions

We investigated the effects of pre-dealloying treatment by cold rolling and annealing the original alloy on the microstructure of NPG produced by dealloying. It was found that plastic deformation and annealing prior to dealloying can lead to reduced ligament and pore sizes of NPG. Moreover, post-deformation annealing can facilitate the formation of continuous, homogeneous, and refined NPG structures. The minima of pore and ligament sizes (both ~8 nm) with the most uniform distribution were obtained in the AN sample with a thickness reduction of 60% and a dealloying time of 7 h. Our study indicated the important role of pre-dealloying treatments of the original alloy (namely plastic deformation and annealing) in the formation and optimization of the NPG microstructure produced by dealloying.

## Figures and Tables

**Table 1:** Average ligament and pore sizes of NPG of CR and AN samples for different thickness reduction.

Parameters	CR Samples				AN Samples			
Thickness reduction	0%	60%	90%	98%	0%	60%	90%	98%
Pore size (nm)	16.1 ± 3.4	13.7 ± 3.8	9.4 ± 2.5	8.0 ± 2.0	10.5 ± 2.0	7.5 ± 1.7	14.1 ± 3.3	12.0 ± 2.4
Ligament size (nm)	20.2 ± 4.7	16.0 ± 2.6	11.5 ± 2.4	8.1 ± 1.5	20.3 ± 4.3	7.9 ± 1.2	17.7 ± 3.0	13.0 ± 2.2
